# Supplementary material for: Stacking Model‐Based Classifiers for Dealing With Multiple Sets of Noisy Labels
Source: Biom J. 2025 Mar 12;67(2):e70042. doi: 10.1002/bimj.70042 (PMC11898607; doi:10.1002/bimj.70042)
Supplement: Supplementary file 1 — Supporting Information [file BIMJ-67-e70042-s001.zip › density-based-ensemble-model-main/competitors_real_data/netlab-master/netlab-master/help/netpak.htm]

Netlab Reference Manual netpak


# netpak

## Purpose

Combines weights and biases into one weights vector.

## Synopsis

```
w = netpak(net)
```

## Description

`w = netpak(net)` takes a network data structure `net` and
combines the component weight matrices into a single row
vector `w`. The facility to switch between these two
representations for the network parameters is useful, for example, in
training a network by error function minimization, since a single
vector of parameters can be handled by general-purpose optimization
routines. This function also takes into account a `mask` defined
as a field in `net` by removing any weights that correspond to
entries of 0 in the mask.

## See Also

`net`, `netunpak`, `netfwd`, `neterr`, `netgrad`

---

**Pages:**
Index


---

Copyright (c) Ian T Nabney (1996-9)
